# Supplementary material for: Predicting mortality from AI cardiac volumes mass and coronary calcium on chest computed tomography
Source: Nat Commun. 2024 Mar 29;15:2747. doi: 10.1038/s41467-024-46977-3 (PMC10980695; doi:10.1038/s41467-024-46977-3)
Supplement: Supplementary file 1 — Supplementary Information [file 41467_2024_46977_MOESM1_ESM.pdf]

SUPPLEMENT

Supplemental Figure 1

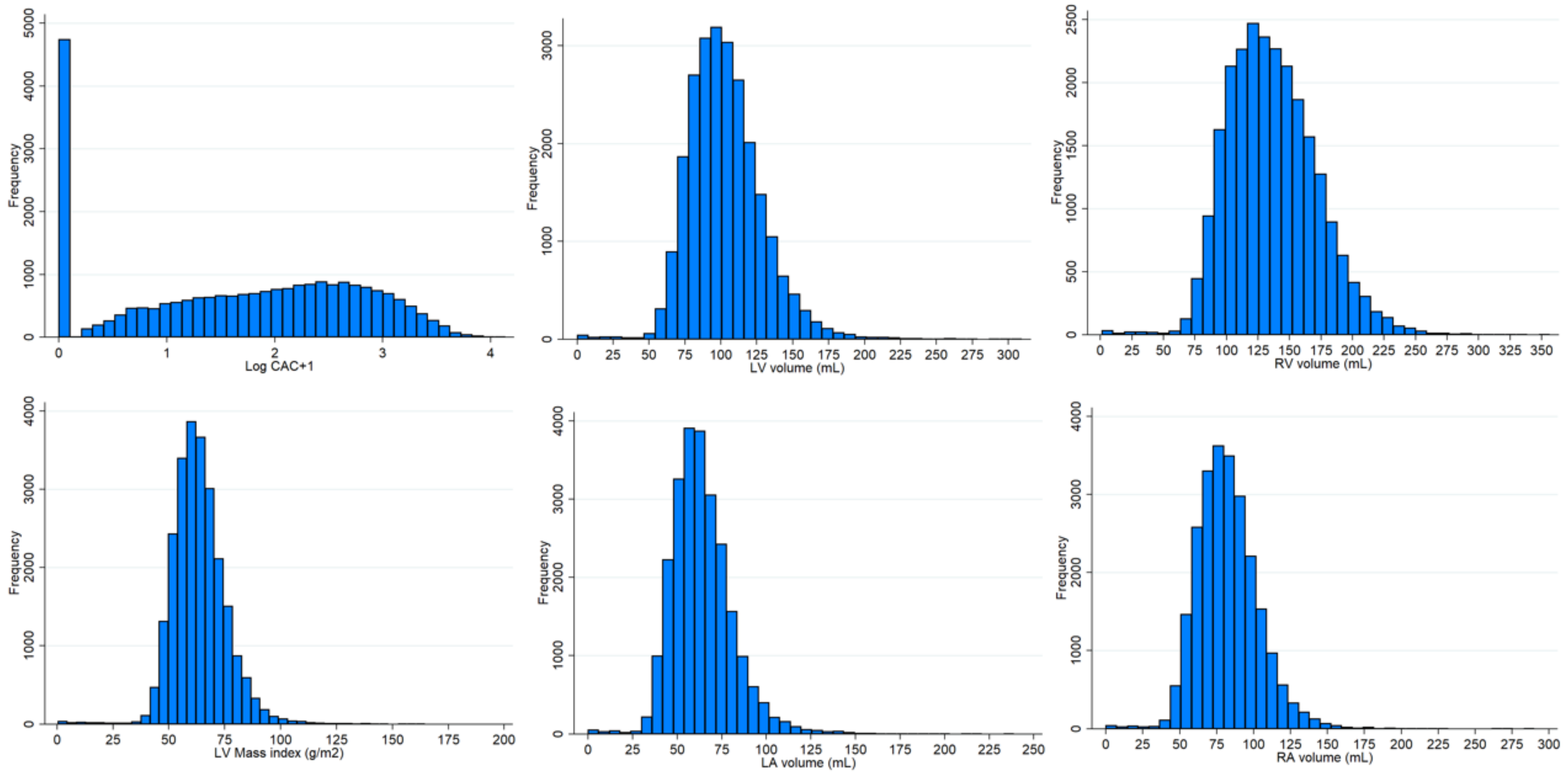

Supplemental Figure 1. Distribution of automated measures. Distribution of coronary artery coronary artery calcium (CAC), left ventricular (LV) mass index, cardiac chamber volumes, shape index, and eccentricity index in blue bars. LA – left atrial, RA – right atrial, RV – right ventricular. Source data is available.

Supplemental Figure 2

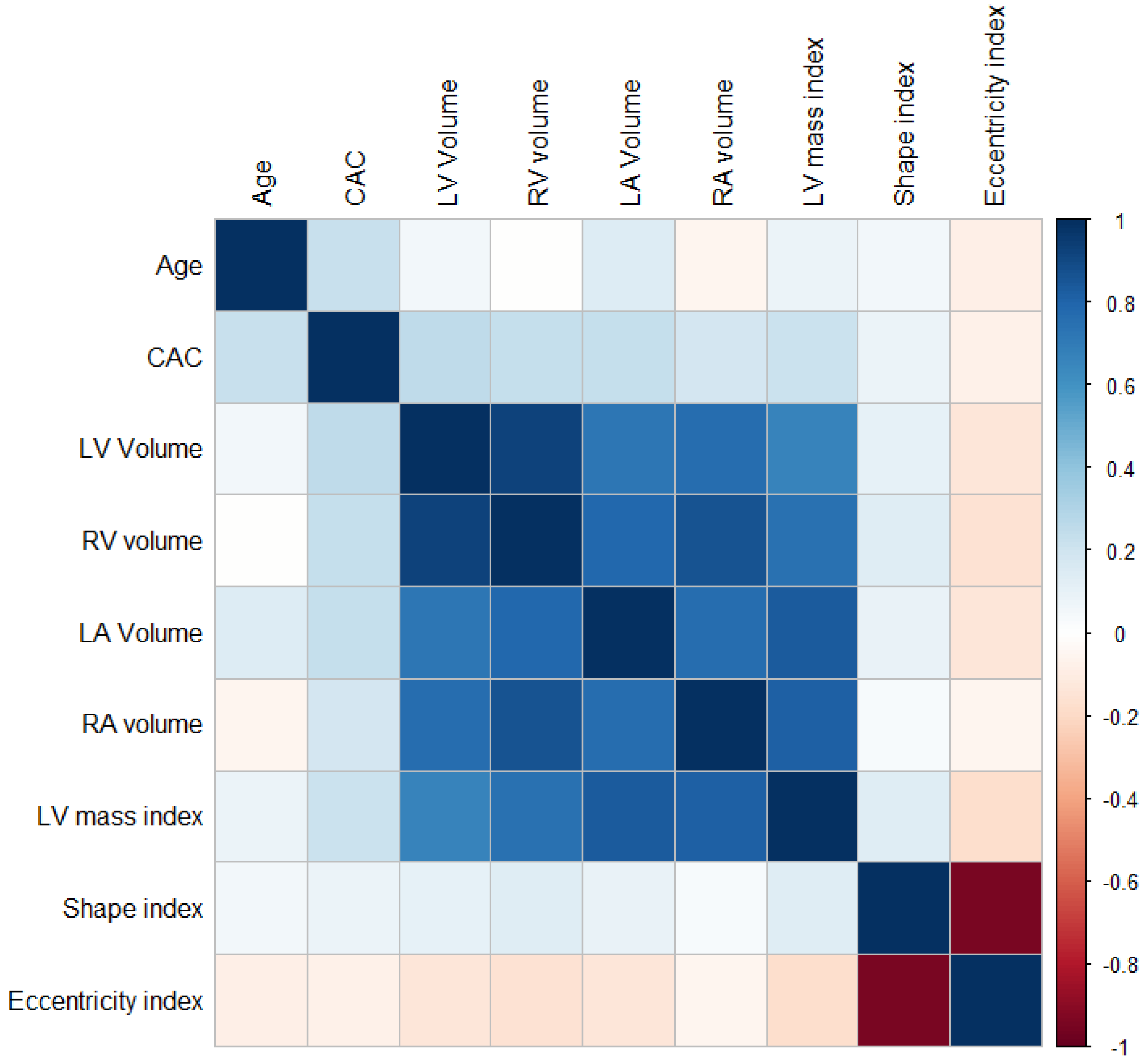

Supplemental Figure 2: Correlation matrix. Correlation matrix for age, coronary artery calcium (CAC), left ventricular (LV) mass index, cardiac chamber volumes, shape index, and eccentricity index. LA – left atrial, RA – right atrial, RV – right ventricular. Colors reflect correlations as outlined in Figure. Source data is available.

Supplemental Figure 3

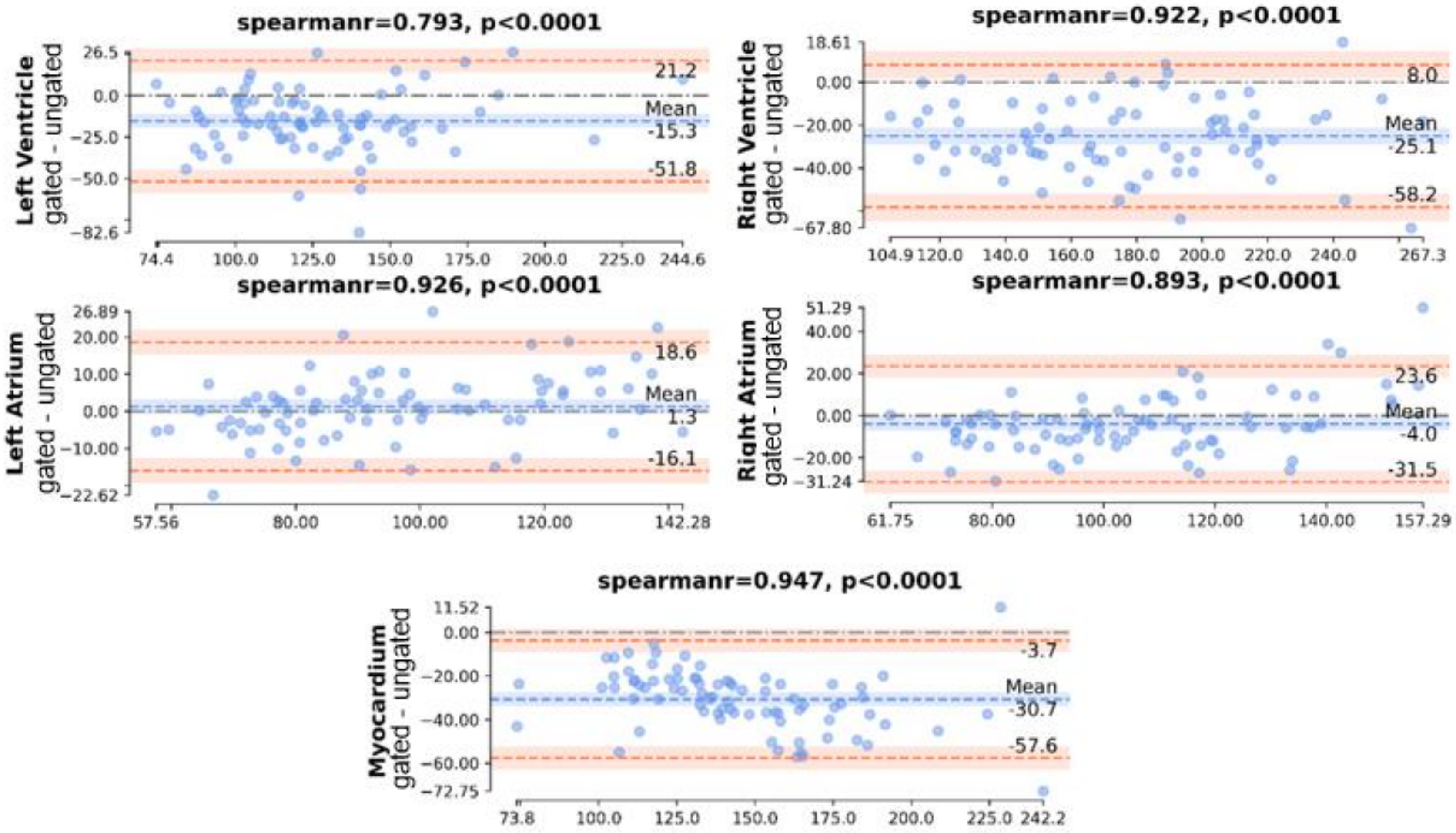

Supplemental Figure 3: Correlation between estimates of cardiac volumes and left ventricular mass from low-dose, ungated CT and contrast-enhanced, ECG-gated, cardiac CT. Individual patients shown as blue dots with limits of agreement with orange dashed lines.

Supplemental Figure 4

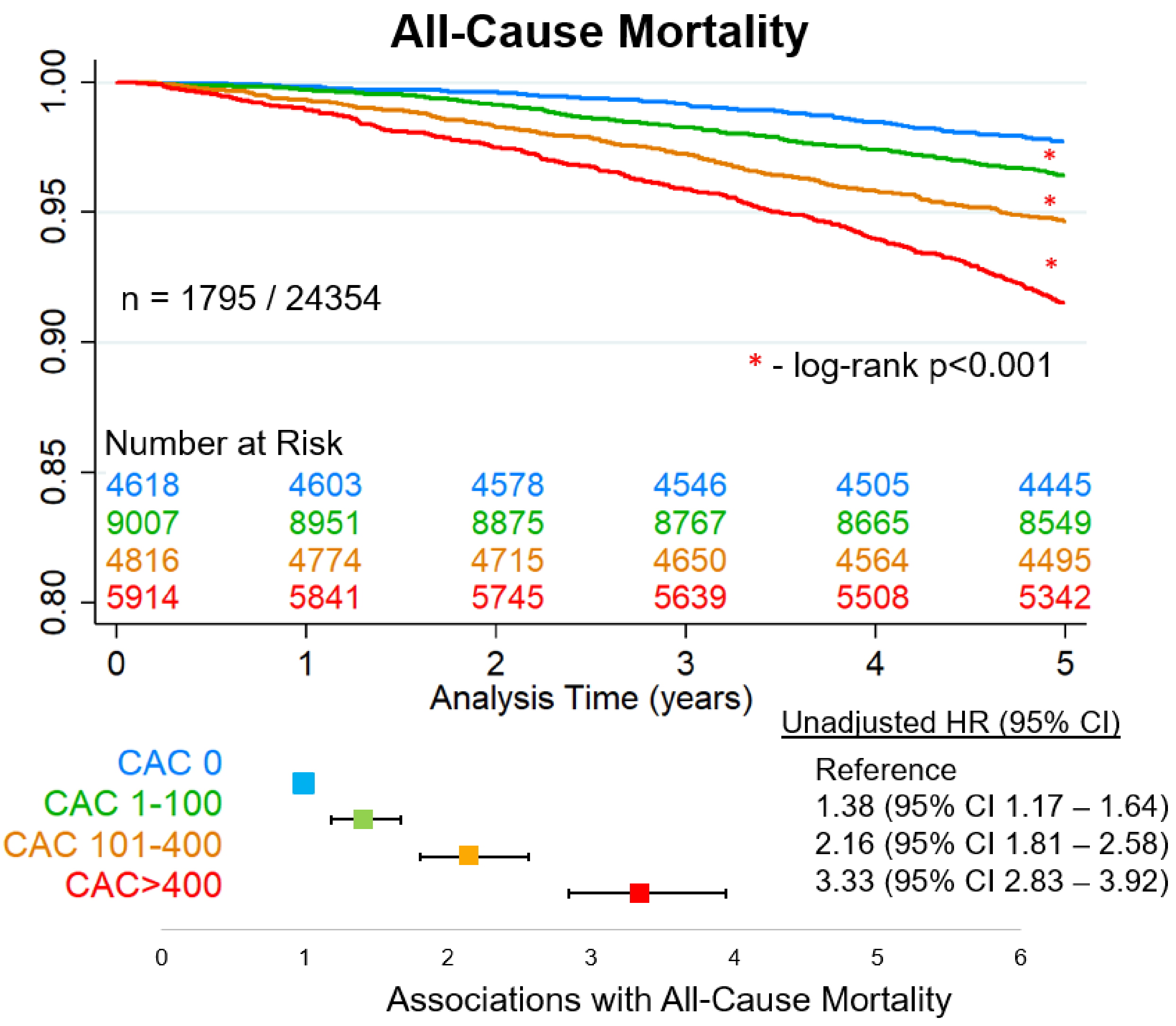

Supplemental Figure 4: Kaplan-Meier survival curves for all-cause mortality patients stratified by deep-learning coronary artery calcium (CAC) scores. Colors reflect CAC score groups as defined in the figure. CI – confidence interval, HR – hazard ratio. Source data is available.

Supplemental Figure 5

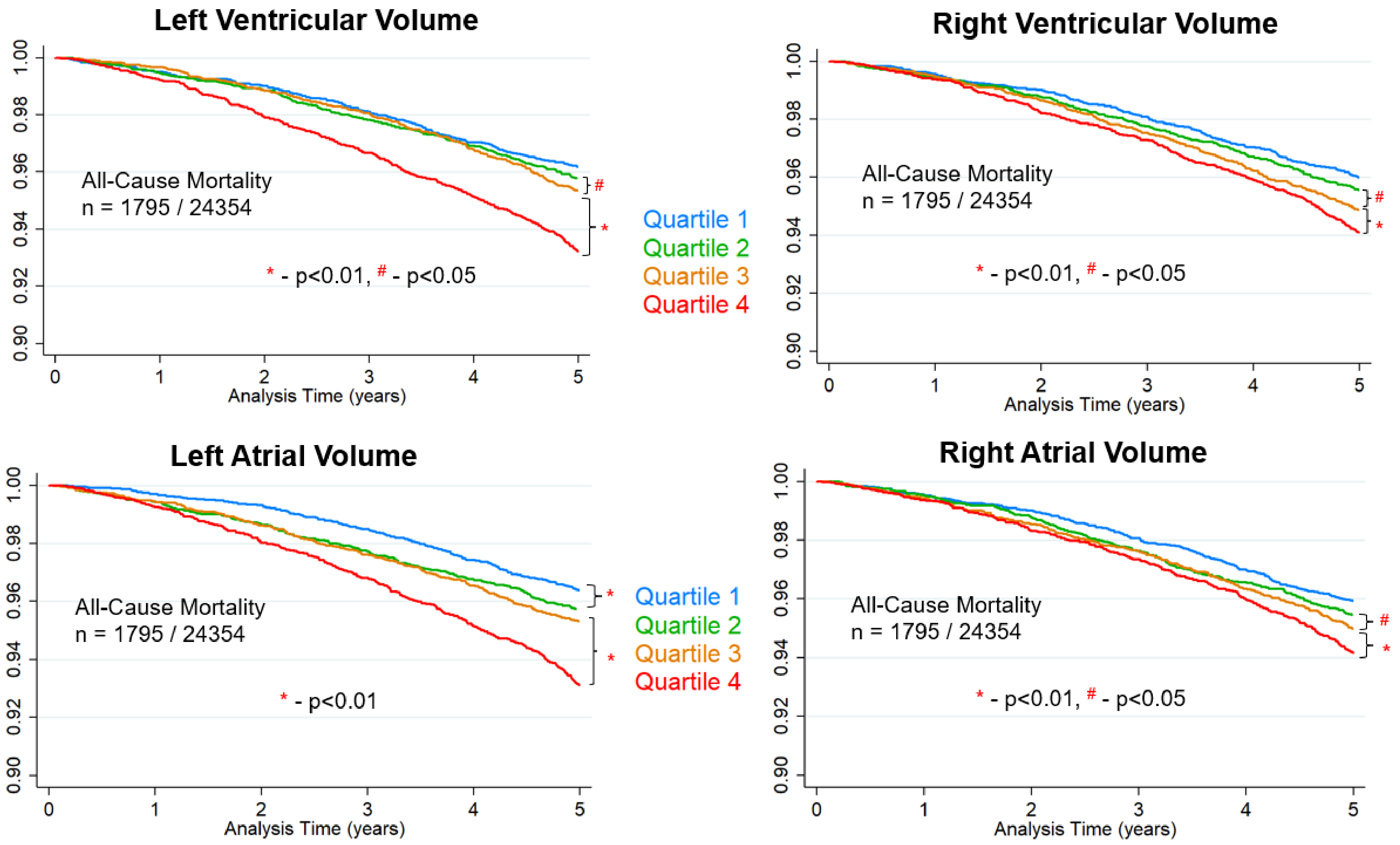

Supplemental Figure 5: Kaplan-Meier survival curves for all-cause mortality stratified by quartile of chamber volume. Colors reflect quartiles as defined in the figure. Source data is available.

Supplemental Figure 6

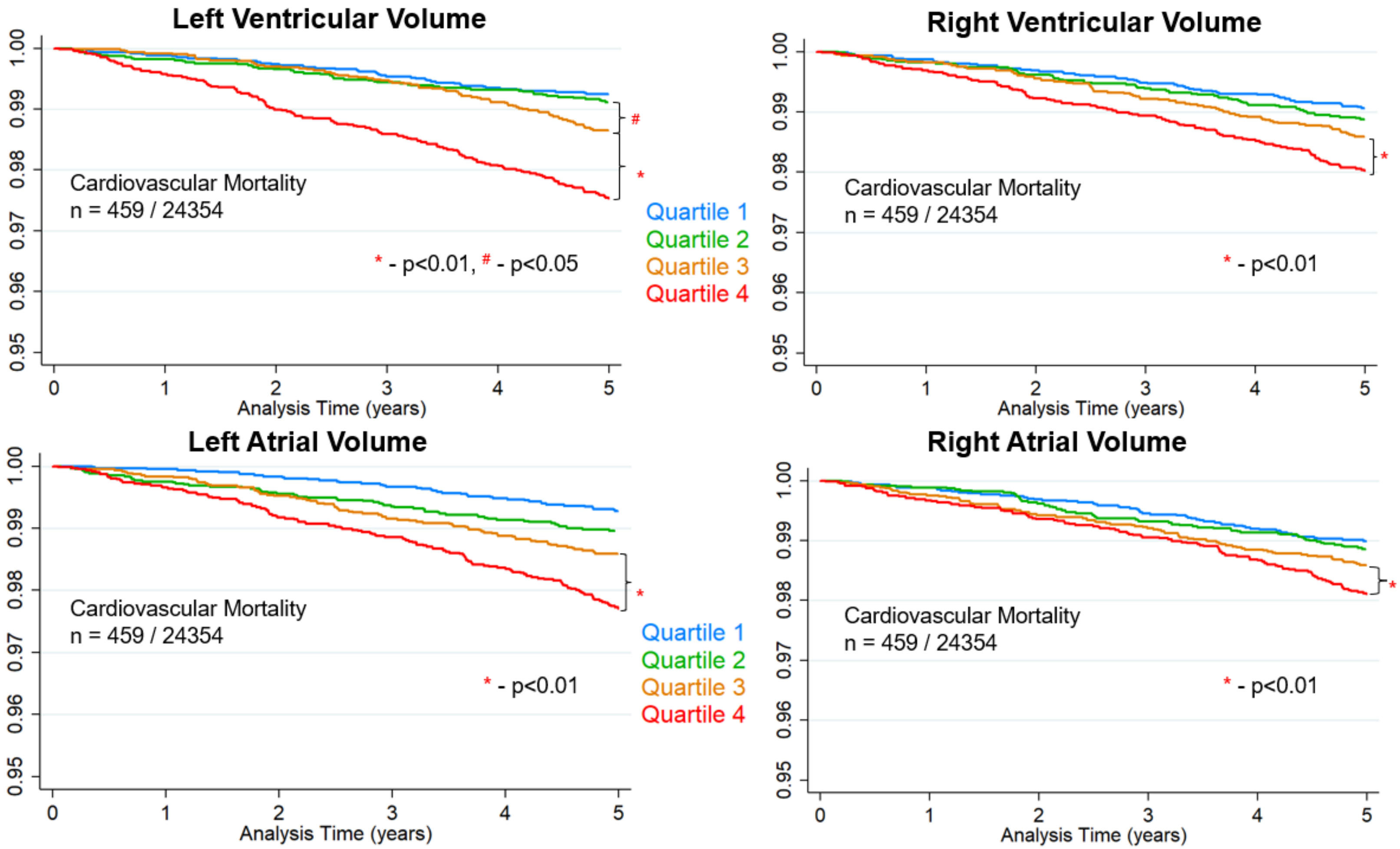

Supplemental Figure 6: Kaplan-Meier survival curves for cardiovascular mortality stratified by quartile of chamber volume. Colors reflect quartiles as defined in the figure. Source data is available.

Supplemental Figure 7

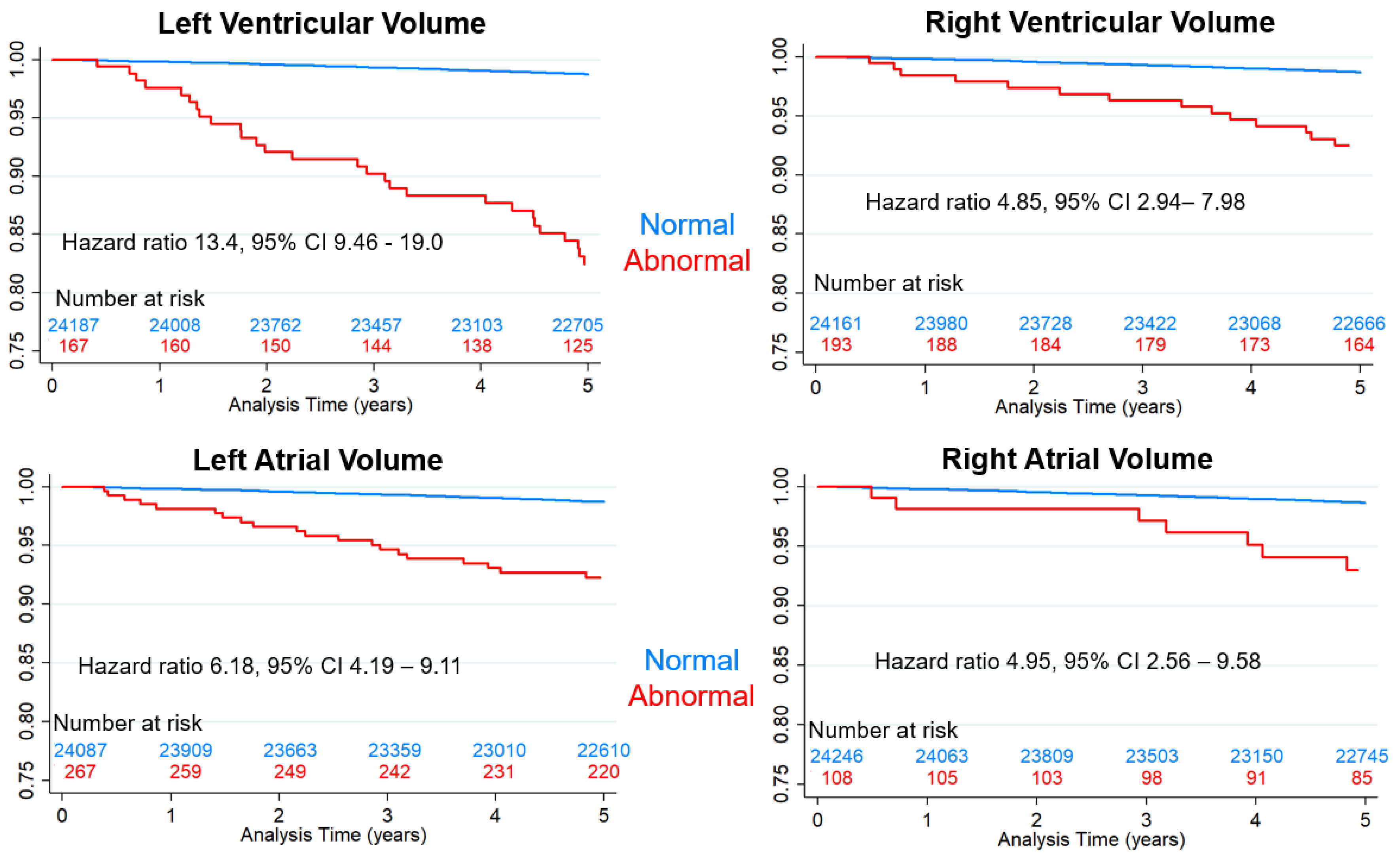

Supplemental Figure 7: Kaplan-Meier survival curves for cardiovascular mortality by normal (blue) or abnormal (red) chamber volume. In women, the thresholds for abnormal chamber volumes were: left ventricle volume > 147mL, right ventricle volume > 180mL, left atrium volume > 99mL and right

atrium volume >126mL. In men, the thresholds for abnormal chamber volumes were: left ventricle volume > 195mL, right ventricle volume > 240mL, left atrium volume > 121mL and right atrium volume >162mL. CI – confidence interval. Source data is available.

Supplemental Figure 8

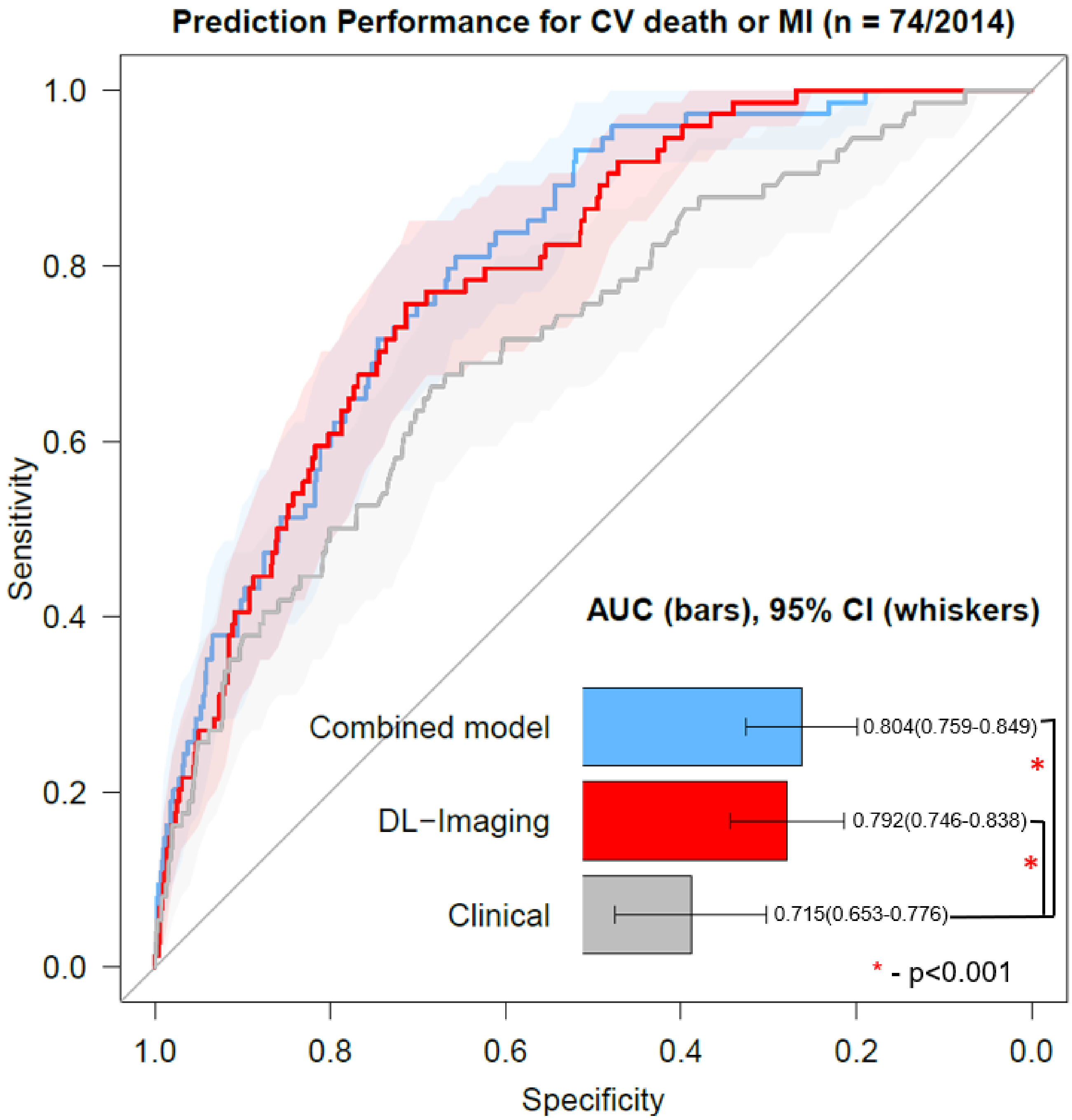

Supplemental Figure 8: Receiver operating characteristic curves for cardiac (CV) death or myocardial infarction (MI) for the Early Identification of Subclinical Atherosclerosis by Noninvasive Imaging Research trial. The models included were clinical (age, sex, medical history - grey), deep learning (DL) derived imaging variables (coronary artery calcium, cardiac volumes, shape index, eccentricity index, and left ventricular mass index - red), and a combined model incorporating all variables (DL derived and clinical variables - blue). Variables were integrated using logistic regression analyses. AUC – area under the receiver operating characteristic curve, CI – confidence interval. Source data is available.

Supplemental Figure 9

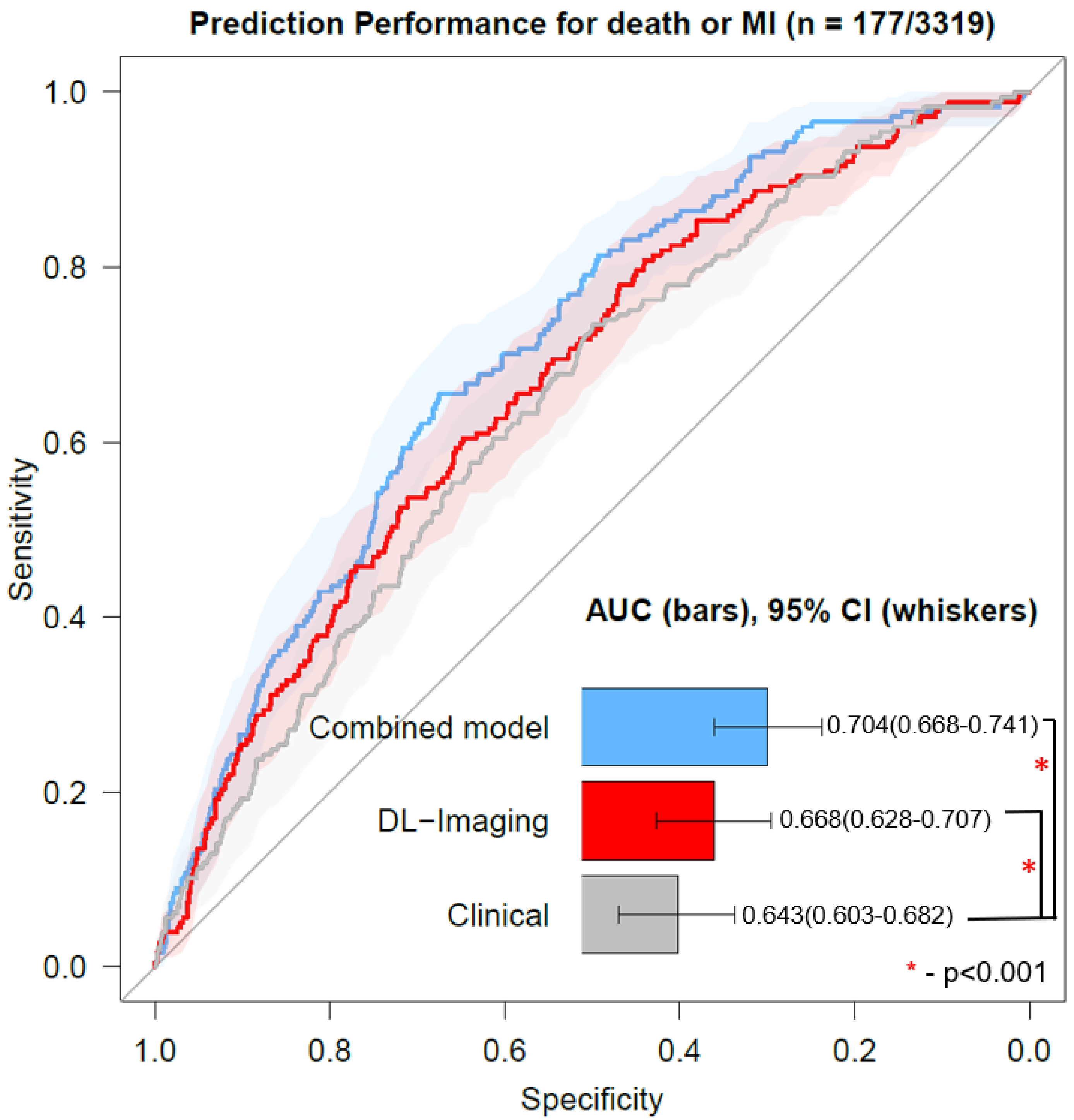

Supplemental Figure 9: Receiver operating characteristic curves for death or myocardial infarction (MI) in the low dose computed tomography population. The models included were clinical (age, sex, medical history - grey), deep learning (DL) derived imaging variables (coronary artery calcium, cardiac volumes, shape index, eccentricity index, and left ventricular mass index - red), and a combined model incorporating all variables (DL derived and clinical variables - blue). Variables were integrated using logistic regression analyses. AUC – area under the receiver operating characteristic curve, CI – confidence interval. Source data is available.

Supplemental Figure 10

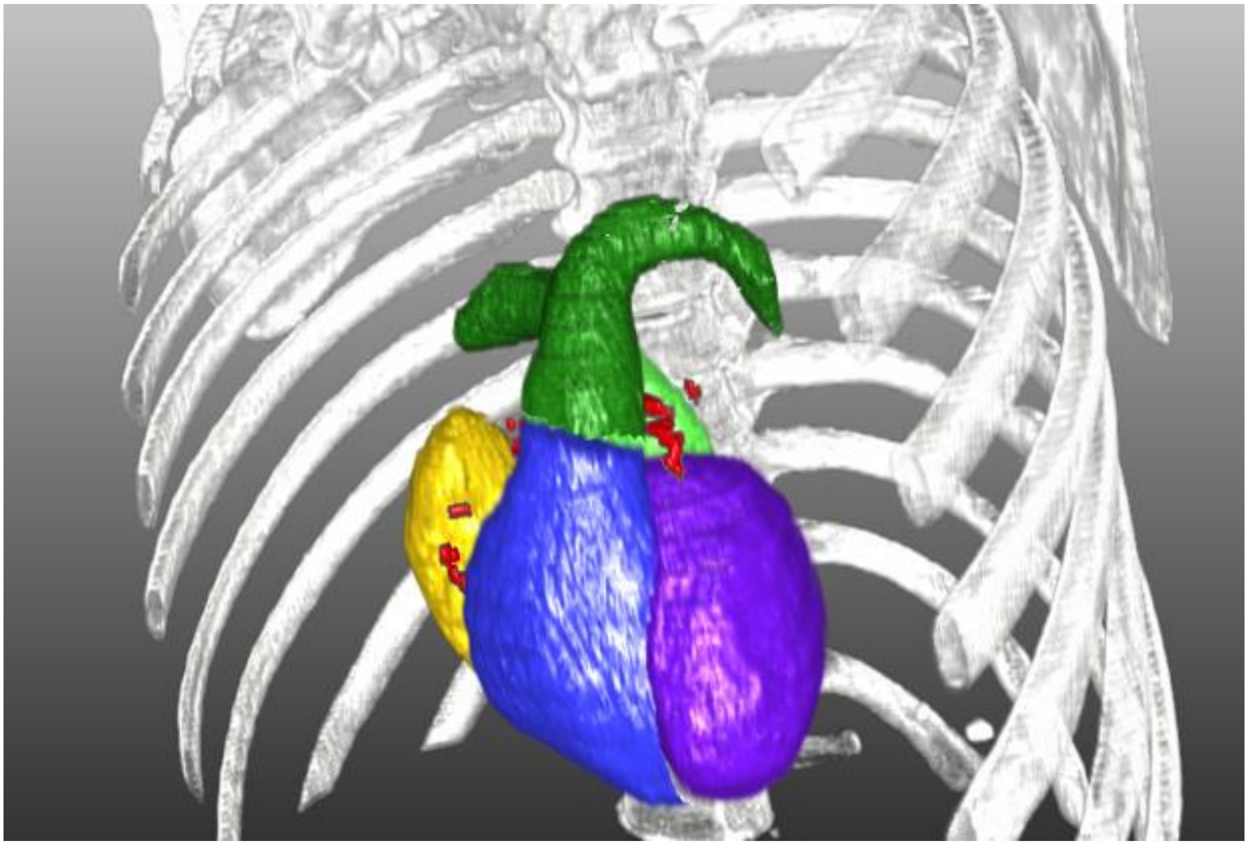

Supplemental Figure 10: Three-dimensional segmented cardiac volumes for a patient. Left ventricular myocardium (purple), right ventricle (blue), right atrium (yellow) , left atrium (light green) were segmented using TotalSegmentator. Coronary calcium (red) was segmented using a convolutional long-short term memory deep learning model.

Supplemental Figure 11

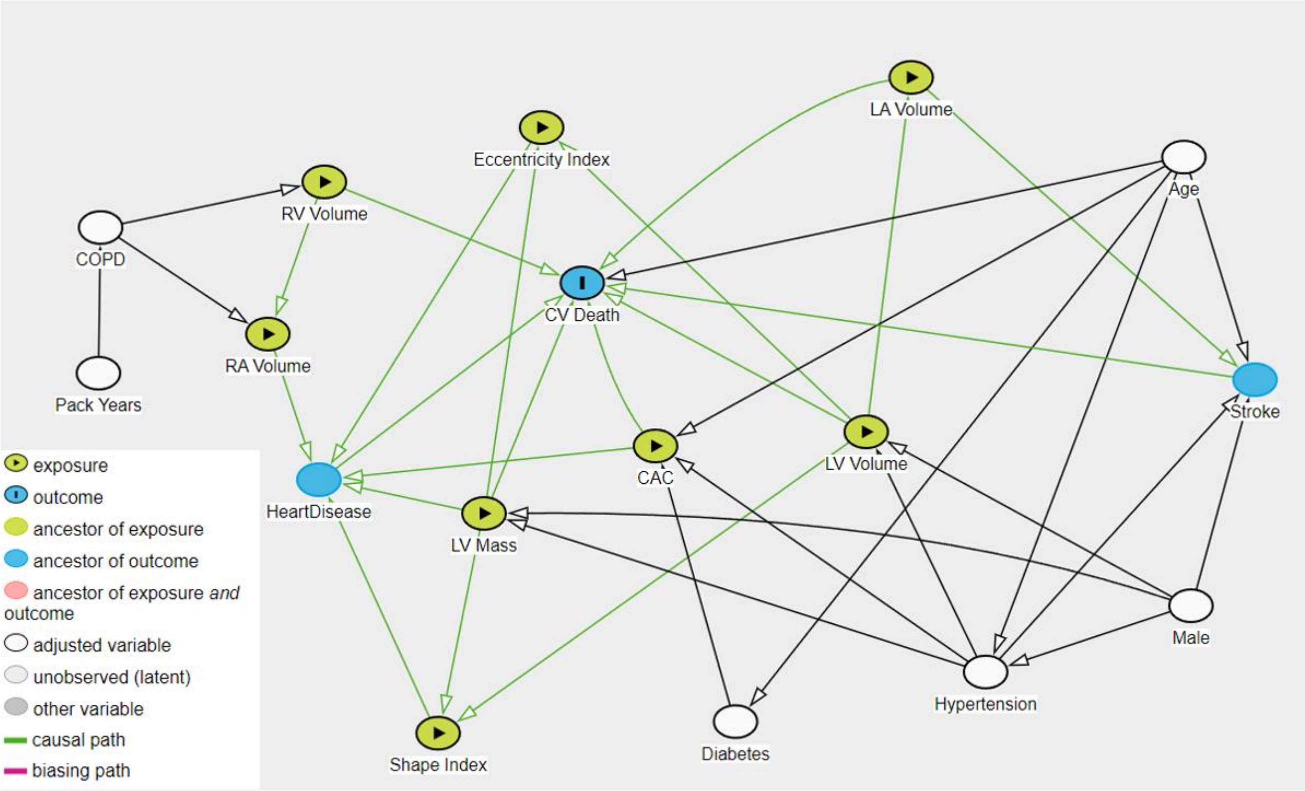

Supplemental Figure 11: Directed acyclic graph outlining suspected relationships between variables and cardiovascular mortality. Colors defined in embedded figure legend. CAC – coronary artery calcium, COPD – chornic obstructive pulmonary disease, CV – cardiovascular, LA – left atrium, LV – left ventricle, RA – right atrium, RV – right ventricle.

Supplemental Table 1

	All-Cause Mortality		Cardiovascular Mortality	
	Unadjusted HR (95% CI)	p-value	Unadjusted subHR (95% CI)	p-value
CAC 0	Reference	--	Reference	--
CAC 1 – 100	1.38 (1.17 – 1.64)	<0.001	1.74 (1.13 – 2.67)	<0.001
CAC 101 – 400	2.16 (1.81 – 2.58)	<0.001	3.62 (2.37 – 5.54)	<0.001
CAC > 400	3.33 (2.83 – 3.92)	<0.001	7.07 (4.75 – 10.5)	<0.001
Left ventricular volume (per 10 mL)	1.09 (1.07 – 1.10)	<0.001	1.15 (1.11 – 1.19)	<0.001
Right ventricular volume (per 10 mL)	1.04 (1.03 – 1.06)	<0.001	1.08 (1.06 – 1.11)	<0.001
Left atrial volume (per 10 mL)	1.13 (1.11 – 1.15)	<0.001	1.18 (1.14 – 1.22)	<0.001
Right atrial volume (per 10 mL)	1.08 (1.06 – 1.10)	<0.001	1.12 (1.09 – 1.15)	<0.001
LV myocardial mass index (per 10 g/m^2)	1.26 (1.23 – 1.30)	<0.001	1.46 (1.38 – 1.54)	<0.001
Shape index (per 0.1)	1.31 (1.20 – 1.43)	<0.001	1.68 (1.40 – 2.01)	<0.001
Eccentricity index (per 0.1)	0.64 (0.55 – 0.74)	<0.001	0.45 (0.34 – 0.61)	<0.001
Age (per 10 years)	2.22 (2.03 – 2.41)	<0.001	2.27 (1.91 – 2.69)	<0.001
Male	1.62 (1.47 – 1.79)	<0.001	1.82 (1.49 – 2.39)	<0.001
Pack years smoking (per 10 years)	1.11 (1.09 – 1.13)	<0.001	1.11 (1.08 – 1.14)	<0.001
Hypertension	1.37 (1.25 – 1.50)	<0.001	1.81 (1.51 – 2.17)	<0.001
Diabetes	1.95 (1.72 – 2.21)	<0.001	2.25 (1.78 – 2.84)	<0.001
Heart Disease	1.95 (1.74 – 2.18)	<0.001	2.84 (2.32 – 3.47)	<0.001
COPD	2.19 (1.88 – 2.55)	<0.001	1.74 (1.26 – 2.42)	0.001
Stroke	2.30 (1.89 – 2.79)	<0.001	3.29 (2.37 – 4.57)	<0.001

Supplemental Table 1: Unadjusted associations with all-cause mortality and cardiovascular mortality. Associations with all-cause mortality evaluated using Cox proportional hazards model and cardiovascular mortality assessed with Fine-Gray competing risk model with no adjustment for multiple comparisons. CAC – coronary artery calcification, CI – confidence interval, COPD – chronic obstructive pulmonary disease, HR – hazard ratio, LV – left ventricle, subHR – subdistribution hazard ratio.

Supplemental Table 2

	Adjusted HR (95% CI)	p-value
CAC 0	Reference	Reference
CAC 1 – 100	1.24 (1.04 - 1.47)	0.015
CAC 101 – 400	1.56 (1.30 - 1.87)	<0.001
CAC > 400	1.88 (1.57 - 2.24)	<0.001
Left ventricular volume (per 10 mL)	0.94 (0.88 - 0.99)	0.024
Right ventricular volume (per 10 mL)	0.95 (0.92 - 0.99)	0.004
Left atrial volume (per 10 mL)	1.11 (1.06 - 1.16)	<0.001
Right atrial volume (per 10 mL)	0.95 (0.91 - 0.99)	0.027
LV mass index (per 10 g/m2)	1.34 (1.22 - 1.47)	<0.001
Shape index (per 0.1)	1.31 (1.02 - 1.66)	0.031
Eccentricity index (per 0.1)	1.31 (0.86 - 1.99)	0.202
Age (per 10 years)	1.59 (1.44 - 1.75)	<0.001
Male	1.28 (1.12 - 1.46)	<0.001
Pack years smoking (per 10 years)	1.06 (1.04 - 1.07)	<0.001
Hypertension	0.99 (0.90 - 1.1)	0.907
Diabetes	1.53 (1.34 - 1.74)	<0.001
Heart disease	1.08 (0.95 - 1.22)	0.223
COPD	1.96 (1.68 - 2.30)	<0.001
Stroke	1.54 (1.26 - 1.88)	<0.001

Supplemental Table 2: Associations with all-cause mortality. Associations evaluated using Cox proportional hazards model with no adjustment for multiple comparisons. CAC – coronary artery calcification, CI – confidence interval, COPD – chronic obstructive pulmonary disease, HR – hazard ratio, LV – left ventricle.

Supplemental Table 3

	No History of Heart Disease		History of Heart Disease	
	Adjusted subHR (95% CI)	p-value	Adjusted subHR (95% CI)	p-value
CAC 0	Reference	--	Reference	--
CAC 1 – 100	1.58 (0.99 - 2.53)	0.054	0.96 (0.31 - 2.94)	0.942
CAC 101 – 400	2.84 (1.76 - 4.59)	<0.001	0.92 (0.31 - 2.76)	0.883
CAC > 400	3.85 (2.39 - 6.21)	<0.001	1.39 (0.50 - 3.87)	0.527
Left ventricular volume (per 10 mL)	1.18 (1.03 - 1.36)	0.018	1.02 (0.87 - 1.2)	0.798
Right ventricular volume (per 10 mL)	0.88 (0.81 - 0.96)	0.002	0.97 (0.86 - 1.11)	0.678
Left atrial volume (per 10 mL)	1.19 (1.07 - 1.32)	0.001	1.08 (0.95 - 1.23)	0.223
Right atrial volume (per 10 mL)	0.86 (0.77 - 0.95)	0.005	0.92 (0.81 - 1.05)	0.217
LV myocardial mass (per 10 g/m^2)	1.17 (0.92 - 1.48)	0.200	1.37 (1.04 - 1.80)	0.025
Shape index (per 0.1)	1.34 (0.70 - 2.53)	0.375	1.92 (0.89 - 4.16)	0.097
Eccentricity index (per 0.1)	1.57 (0.52 - 4.80)	0.426	1.90 (0.49 - 7.34)	0.353
Age (per 10 years)	1.56 (1.24 - 1.96)	<0.001	1.13 (0.77 - 1.65)	0.547
Male	1.16 (0.85 - 1.56)	0.350	0.91 (0.54 - 1.52)	0.710
Pack years smoking (per 10 years)	1.04 (1.00 - 1.08)	0.048	1.04 (0.97 - 1.11)	0.307
Hypertension	1.16 (0.92 - 1.47)	0.214	0.94 (0.66 - 1.36)	0.756
Diabetes	1.22 (0.87 - 1.71)	0.247	1.68 (1.14 - 2.47)	0.009
COPD	1.79 (1.19 - 2.72)	0.006	1.37 (0.75 - 2.49)	0.308
Stroke	2.27 (1.49 - 3.45)	<0.001	1.45 (0.79 - 2.64)	0.228

Supplemental Table 3: Associations with cardiovascular mortality in patients with and without a history of heart disease from multivariable analyses. Associations evaluated using Fine-Gray competing risk model with no adjustment for multiple comparisons. CAC – coronary artery calcification, CI – confidence interval, COPD – chronic obstructive pulmonary disease, LV – left ventricle, subHR – subhazard ratio.

Supplemental Table 4

	Adjusted subHR (95% CI)	p-value
CAC 0	Reference	Reference
CAC 1 – 100	1.50 (0.96 - 2.34)	0.072
CAC 101 – 400	2.49 (1.58 - 3.93)	<0.001
CAC > 400	3.53 (2.24 - 5.56)	<0.001
Left ventricular volume (per 10 mL)	1.16 (1.03 - 1.30)	0.014
Right ventricular volume (per 10 mL)	0.89 (0.83 - 0.97)	0.005
Left atrial volume (per 10 mL)	1.13 (1.03 - 1.24)	0.01
Right atrial volume (per 10 mL)	0.89 (0.81 - 0.98)	0.013
LV myocardial mass (per 10 g/m^2)	1.23 (1.02 - 1.49)	0.033
Shape index (per 0.1)	1.49 (0.85 - 2.61)	0.168
Eccentricity index (per 0.1)	1.73 (0.63 - 4.72)	0.287
Age (per 10 years)	1.37 (1.11 - 1.70)	0.003
Male	1.14 (0.86 - 1.52)	0.358
Pack years smoking (per 10 years)	1.06 (1.02 - 1.09)	0.002
Hypertension	1.14 (0.92 - 1.41)	0.235
Diabetes	1.35 (1.02 - 1.77)	0.034
Heart disease	1.06 (0.83 - 1.37)	0.628
COPD	1.76 (1.23 - 2.52)	0.002
Stroke	2.03 (1.40 - 2.94)	<0.001

Supplemental Table 4: Associations with cardiovascular mortality in patients without reported cardiovascular abnormalities from multivariable model. Associations evaluated using Fine-Gray competing risk model with no adjustment for multiple comparisons. CAC – coronary artery calcification, CI – confidence interval, COPD – chronic obstructive pulmonary disease, LV – left ventricle, subHR – subhazard ratio.

Supplemental Table 5

	Tube voltage 120 kVp n=394/21287		Tube voltage 140 kVp n=58/2313	
	Unadjusted subHR (95% CI)	p-value	Unadjusted subHR (95% CI)	p-value
CAC 0	Reference	--	Reference	--
CAC 1 – 100	2.07 (1.26 – 3.37)	0.004	0.81 (0.32 – 2.02)	0.648
CAC 101 – 400	4.34 (2.67 – 7.06)	<0.001	1.44 (0.56 – 3.70)	0.454
CAC > 400	8.40 (5.31 – 13.3)	<0.001	3.12 (1.36 – 7.17)	0.007
Left ventricular volume (per 10 mL)	1.15 (1.11 – 1.19)	<0.001	1.17 (1.09 – 1.25)	<0.001
Right ventricular volume (per 10 mL)	1.08 (1.06 – 1.11)	<0.001	1.09 (1.01 – 1.16)	0.018
Left atrial volume (per 10 mL)	1.17 (1.13 – 1.22)	<0.001	1.20 (1.08 – 1.34)	0.001
Right atrial volume (per 10 mL)	1.12 (1.09 – 1.15)	<0.001	1.10 (0.98 – 1.23)	0.099
LV myocardial mass (per 10 g/m^2)	1.46 (1.37 – 1.55)	<0.001	1.43 (1.27 – 1.60)	<0.001
Shape index (per 0.1)	1.64 (1.35 – 1.99)	<0.001	2.27 (1.33 – 3.90)	0.001
Eccentricity index (per 0.1)	0.46 (0.34 – 0.64)	<0.001	0.32 (0.14 – 0.72)	0.004

Supplemental Table 5: Associations with cardiovascular mortality stratified by tube voltage. There were no statistically significant interactions between tube voltage category and risk from imaging variables (all interaction p-values>0.05 after Bonferroni correction). Associations evaluated using Fine-Gray competing risk model, only interaction tests were adjusted multiple comparisons. CAC – coronary artery calcification, CI – confidence interval, COPD – chronic obstructive pulmonary disease, LV – left ventricle, subHR – subhazard ratio.

Supplemental Table 6

	Slice thickness<2.5mm n=199/9443		Slice thickness 2.5mm n=207/11417		Slice thickness>2.5mm n=53/3494	
	Unadjusted subHR (95% CI)	p-value	Unadjusted subHR (95% CI)	p-value	Unadjusted subHR (95% CI)	p-value
CAC 0	Reference	--	Reference	--	Reference	--
CAC 1 – 100	3.07 (1.30 – 7.22)	0.010	1.19 (0.69 – 2.06)	0.527	2.57 (0.56 – 11.7)	0.223
CAC 101 – 400	5.38 (2.28 – 12.7)	<0.001	2.63 (1.53 – 4.51)	<0.001	7.47 (1.73 – 32.3)	0.007
CAC > 400	11.1 (4.87 – 25.2)	<0.001	5.62 (3.45 – 9.15)	<0.001	8.98 (2.12 – 38.0)	0.003
Left ventricular volume (per 10 mL)	1.20 (1.15 – 1.25)	<0.001	1.13 (1.09 – 1.17)	<0.001	1.24 (1.16 – 1.33)	<0.001
Right ventricular volume (per 10 mL)	1.10 (1.06 – 1.13)	<0.001	1.07 (1.04 – 1.10)	<0.001	1.14 (1.05 – 1.23)	<0.001
Left atrial volume (per 10 mL)	1.16 (1.11 – 1.22)	<0.001	1.17 (1.12 – 1.23)	<0.001	1.35 (1.23 – 1.48)	<0.001
Right atrial volume (per 10 mL)	1.11 (1.07 – 1.16)	<0.001	1.11 (1.06 – 1.16)	<0.001	1.22 (1.08 – 1.37)	<0.001
LV myocardial mass (per 10 g/m^2)	1.50 (1.38 – 1.64)	<0.001	1.42 (1.32 – 1.53)	<0.001	1.52 (1.33 – 1.75)	<0.001
Shape index (per 0.1)	1.45 (1.10 – 1.92)	0.009	2.16 (1.68 – 2.75)	<0.001	0.86 (0.52 – 1.41)	0.556
Eccentricity index (per 0.1)	0.57 (0.36 – 0.91)	0.017	0.31 (0.21 – 0.47)	<0.001	1.19 (0.53 – 2.68)	0.677

Supplemental Table 6: Associations with cardiovascular mortality stratified by slice thickness. There were no statistically significant interactions between slice thickness and risk from imaging variables (all interaction p--values>0.05 after Bonferroni correction). Associations evaluated using Fine-Gray competing risk model, only interaction tests were adjusted multiple comparisons. CAC – coronary artery calcification, CI – confidence interval, COPD – chronic obstructive pulmonary disease, LV – left ventricle, subHR – subhazard ratio.

Supplemental Table 7

Coronary Artery Calcium

Without Events	Low (<0.5%/year)	Intermediate (0.5-1%/year)	High (≥1%/year)		
Low (<0.5%/year)	19114	3250	26	Decreased Risk	0.027
Intermediate (0.5-1%/year)	646	793	66	Increased Risk	0.140
High (≥1%/year)	0	0	0	Net Reclassification	-0.113

With Events	Low (<0.5%/year)	Intermediate (0.5-1%/year)	High (≥1%/year)		
Low (<0.5%/year)	242	159	2	Decreased Risk	0.039
Intermediate (0.5-1%/year)	18	36	2	Increased Risk	0.355
High (≥1%/year)	0	0	0	Net Reclassification	0.316
		**Overall Reclassification (95% CI)**		**0.203 (0.153 – 0.253)**	

Cardiac Volumes

Without Events	Low (<0.5%/year)	Intermediate (0.5-1%/year)	High (≥1%/year)		
Low (<0.5%/year)	21100	1025	265	Decreased Risk	0.044
Intermediate (0.5-1%/year)	1049	348	108	Increased Risk	0.059
High (≥1%/year)	0	0	0	Net Reclassification	-0.015

With Events	Low (<0.5%/year)	Intermediate (0.5-1%/year)	High (≥1%/year)		
Low (<0.5%/year)	297	60	46	Decreased Risk	0.065
Intermediate (0.5-1%/year)	30	14	12	Increased Risk	0.257
High (≥1%/year)	0	0	0	Net Reclassification	0.192
		**Overall Reclassification (95% CI)**		**0.177 (0.128 – 0.226)**	

Shape/Eccentricity Index

Without Events	Low (<0.5%/year)	Intermediate (0.5-1%/year)	High (≥1%/year)		
Low (<0.5%/year)	22147	238	5	Decreased Risk	0.026
Intermediate (0.5-1%/year)	614	864	27	Increased Risk	0.011
High (≥1%/year)	0	0	0	Net Reclassification	0.014

With Events	Low (<0.5%/year)	Intermediate (0.5-1%/year)	High (≥1%/year)		
Low (<0.5%/year)	385	17	1	Decreased Risk	0.031
Intermediate (0.5-1%/year)	14	40	2	Increased Risk	0.044
High (≥1%/year)	0	0	0	Net Reclassification	0.013
		**Overall Reclassification (95% CI)**		**0.028 (0.0003 – 0.053)**	

LV Mass Index

Without Events	Low (<0.5%/year)	Intermediate (0.5-1%/year)	High (≥1%/year)		
Low (<0.5%/year)	20974	1225	191	Decreased Risk	0.041
Intermediate (0.5-1%/year)	974	433	98	Increased Risk	0.063
High (≥1%/year)	0	0	0	Net Reclassification	-0.023

With Events	Low (<0.5%/year)	Intermediate (0.5-1%/year)	High (≥1%/year)		
Low (<0.5%/year)	315	48	40	Decreased Risk	0.059
Intermediate (0.5-1%/year)	27	17	12	Increased Risk	0.218
High (≥1%/year)	0	0	0	Net Reclassification	0.159
		**Overall Reclassification (95% CI)**		**0.136 (0.090 – 0.183)**	

Combined Model

Without Events	Low (<0.5%/year)	Intermediate (0.5-1%/year)	High (≥1%/year)		
Low (<0.5%/year)	19959	1929	502	Decreased Risk	0.040
Intermediate (0.5-1%/year)	952	411	73	Increased Risk	0.105
High (≥1%/year)	0	0	0	Net Reclassification	-0.065
With Events	Low (<0.5%/year)	Intermediate (0.5-1%/year)	High (≥1%/year)		
Low (<0.5%/year)	241	86	76	Decreased Risk	0.059
Intermediate (0.5-1%/year)	27	14	15	Increased Risk	0.386
High (≥1%/year)	0	0	0	Net Reclassification	0.327
		**Overall Reclassification (95% CI)**		**0.259 (0.206 – 0.312)**	

Supplemental Table 7: Net-reclassification analysis for cardiovascular mortality of coronary artery calcium (CAC), cardiac chamber volumes, shape and eccentricity index, left ventricular (LV) mass index, and the combination of all measurements when considered in addition to cardiovascular abnormality identified by a radiologist. Variables were modeled as continuous variables and combined using logistic regression analyses. Red shows patients reclassified to higher risk by one (light) or two (dark) categories, with blue showing patients recategorized to lower risk by one (light) or two (dark) categories. CI – confidence interval.

Supplemental Table 8

Coronary Artery Calcium	Low (<0.5%/year)	Intermediate (0.5-1%/year)	High (≥1%/year)		
Low (<0.5%/year)	19645	1396	3	Decreased Risk	0.031
Intermediate (0.5-1%/year)	599	1468	267	Increased Risk	0.070
High (≥1%/year)	26	123	368	Net Reclassification	-0.038
	Low (<0.5%/year)	Intermediate (0.5-1%/year)	High (≥1%/year)		
Low (<0.5%/year)	247	69	0	Decreased Risk	0.044
Intermediate (0.5-1%/year)	13	60	26	Increased Risk	0.207
High (≥1%/year)	1	6	37	Net Reclassification	0.163
		**Overall Reclassification (95% CI)**		**0.125 (0.0582 – 0.168)**	

Cardiac Volumes	Low (<0.5%/year)	Intermediate (0.5-1%/year)	High (≥1%/year)		
Low (<0.5%/year)	20213	708	123	Decreased Risk	0.057
Intermediate (0.5-1%/year)	1125	943	266	Increased Risk	0.046
High (≥1%/year)	29	199	289	Net Reclassification	0.011
	Low (<0.5%/year)	Intermediate (0.5-1%/year)	High (≥1%/year)		
Low (<0.5%/year)	248	45	23	Decreased Risk	0.072
Intermediate (0.5-1%/year)	23	45	31	Increased Risk	0.216
High (≥1%/year)	2	8	34	Net Reclassification	0.144
		**Overall Reclassification (95% CI)**		**0.155 (0.107 – 0.202)**	

Shape/Eccentricity Index	Low (<0.5%/year)	Intermediate (0.5-1%/year)	High (≥1%/year)		
Low (<0.5%/year)	20584	460	0	Decreased Risk	0.022
Intermediate (0.5-1%/year)	418	1777	139	Increased Risk	0.025
High (≥1%/year)	0	102	415	Net Reclassification	-0.003
	Low (<0.5%/year)	Intermediate (0.5-1%/year)	High (≥1%/year)		
Low (<0.5%/year)	302	14	0	Decreased Risk	0.026
Intermediate (0.5-1%/year)	8	72	19	Increased Risk	0.072
High (≥1%/year)	0	4	40	Net Reclassification	0.046
		**Overall Reclassification (95% CI)**		**0.042 (0.014 – 0.071)**	

LV Mass Index	Low (<0.5%/year)	Intermediate (0.5-1%/year)	High (≥1%/year)		
Low (<0.5%/year)	20244	706	94	Decreased Risk	0.052
Intermediate (0.5-1%/year)	1036	1058	240	Increased Risk	0.044
High (≥1%/year)	13	196	308	Net Reclassification	0.009
	Low (<0.5%/year)	Intermediate (0.5-1%/year)	High (≥1%/year)		
Low (<0.5%/year)	257	41	18	Decreased Risk	0.070
Intermediate (0.5-1%/year)	25	47	27	Increased Risk	0.187
High (≥1%/year)	1	6	37	Net Reclassification	0.118
		**Overall Reclassification (95% CI)**		**0.126 (0.081 – 0.172)**	

Combined Model	Low (<0.5%/year)	Intermediate (0.5-1%/year)	High (≥1%/year)		
Low (<0.5%/year)	20141	714	56	Decreased Risk	0.040
Intermediate (0.5-1%/year)	788	1305	247	Increased Risk	0.043
High (≥1%/year)	0	156	488	Net Reclassification	-0.003

	Low (<0.5%/year)	Intermediate (0.5-1%/year)	High (≥1%/year)		
Low (<0.5%/year)	236	27	5	Decreased Risk	0.059
Intermediate (0.5-1%/year)	15	64	21	Increased Risk	0.115
High (≥1%/year)	0	12	79	Net Reclassification	0.057
		**Overall Reclassification (95% CI)**		**0.054 (0.016 – 0.092)**	

Supplemental Table 8: Net-reclassification analysis for cardiovascular mortality of coronary artery calcium (CAC), cardiac chamber volumes, shape and eccentricity index, left ventricular (LV) mass index, and the combination of all measurements when considered in addition to a multivariable model including age, sex, smoking history, and past medical history. Variables were modeled as continuous variables and combined using logistic regression analyses. Red shows patients reclassified to higher risk by one (light) or two (dark) categories, with blue showing patients recategorized to lower risk by one (light) or two (dark) categories. CI – confidence interval.

Supplemental Table 9

	No events (N=1940)	Cardiac death or myocardial infarction (n=74)	p-value
Age	56 (49, 62)	60 (50, 72)	<0.001
Male	1140 (58.8%)	50 (67.6%)	0.131
Past Medical History			
Hypertension	765 (39.4%)	46 (62.2%)	<0.001
Diabetes	108 (5.6%)	5 (6.8%)	0.660
Dyslipidemia	1,344 (69.3%)	52 (70.3%)	0.899
Family history of CAD	583 (30.1%)	27 (36.5%)	0.242
Smoking	117 (6.0%)	6 (8.1%)	0.461
CAC Category			<0.001
CAC 0	890 (45.9%)	10 (13.5%)	
CAC 1-100	707 (36.4%)	23 (31.1%)	
CAC 101-400	218 (11.2%)	21 (28.4%)	
CAC>400	125 (6.4%)	20 (27.0%)	
LV volume (mL)	95.6 (79.2, 114.2)	100.3 (88.2, 116.1)	0.055
LA volume (mL)	60.6 (51.7, 72.1)	67.9 (60.4, 78.9)	<0.001
RV volume (mL)	124.2 (97.1, 153.5)	125.5 (100.6, 147.8)	0.700
RA volume (mL)	78.7 (67.4, 92.2)	86.5 (75.1, 102.3)	<0.001
LV mass (g)	106.3 (86.1, 126.5)	114.1(98.4, 129.5)	0.016
Shape Index	0.79 (0.75, 0.83)	0.78 (0.74, 0.83)	0.893
Eccentricity Index	0.77 (0.74, 0.80)	0.77 (0.72, 0.80)	0.222

Supplemental Table 9. Population characteristics for the Early Identification of Subclinical Atherosclerosis by Noninvasive Imaging Research trial. Groups were compared using $\chi 2$ tests for categorical variables and Mann-Whitney U test for continuous variables. CAC – coronary artery calcification, CAD – coronary artery disease, LA – left atrium, LV – left ventricle, RA – right atrium, RV – right ventricle.

Supplemental Table 10

	Unadjusted HR (95% CI)	p-value	Adjusted HR (95% CI)	p-value
CAC 0	Reference		Reference	
CAC 1 – 100	2.83 (1.35 – 5.95)	0.006	2.44 (1.16 – 5.16)	0.019
CAC 101 – 400	8.29 (3.90 – 17.6)	<0.001	5.82 (2.68 – 12.7)	<0.001
CAC > 400	13.0 (6.10 – 27.8)	<0.001	7.64 (3.41 – 17.1)	<0.001
Left ventricular volume (per 10 mL)	1.08 (1.00 – 1.17)	0.058	--	
Right ventricular volume (per 10 mL)	1.00 (0.94 – 1.05)	0.898	0.84 (0.74 – 0.95)	0.005
Left atrial volume (per 10 mL)	1.33 (1.20 – 1.47)	<0.001	--	
Right atrial volume (per 10 mL)	1.20 (1.09 – 1.33)	<0.001	1.20 (1.05 – 1.38)	0.007
LV myocardial mass (per 10 g/m^2)	1.18 (1.00 – 1.40)	0.052	1.56 (1.10 – 2.20)	0.012
Shape index (per 0.1)	0.96 (0.66 – 1.40)	0.838	--	
Eccentricity index (per 0.1)	0.63 (0.38 – 1.05)	0.075	--	
Age (per 10 years)	2.13 (1.65 – 2.76)	<0.001	1.37 (1.02 – 1.86)	0.038
Male	1.43 (0.88 – 2.33)	0.150	--	
Hypertension	2.53 (1.58 – 4.05)	<0.001	--	
Diabetes	1.26 (0.51 – 3.11)	0.623	--	
Dyslipidemia	1.05 (0.64 – 1.73)	0.840	--	
Smoking	1.36 (0.59 – 3.12)	0.475	--	
Family history	1.35 (0.84 – 2.17)	0.211	--	

Supplemental Table 10: Associations with cardiovascular death or myocardial infarction in the population from the Early Identification of Subclinical Atherosclerosis by Noninvasive Imaging Research trial. Given the limited number of events, a parsimonious multivariable model was developed using backward stepwise elimination until only variables with significant associations remained. Associations evaluated using Cox proportional hazards model, to account for multiple comparisons, p<0.001 is considered statistically significant. CAC – coronary artery calcium, CI – confidence interval, HR – hazard ratio

Supplemental Table 11

Without Events	Low (<0.25%/year)	Intermediate (0.25-0.5%/year)	High (≥0.5%/year)		
Low (<0.25%/year)	1101	87	48	Decreased Risk	0.178
Intermediate (0.25-0.5%/year)	239	135	111	Increased Risk	0.127
High (≥0.5%/year)	46	61	112	Net Reclassification	0.052
**With Events**	**Low (<0.25%/year)**	**Intermediate (0.25-0.5%/year)**	**High (≥0.5%/year)**		
Low (<0.25%/year)	242	159	2	Decreased Risk	0.135
Intermediate (0.25-0.5%/year)	18	36	2	Increased Risk	0.270
High (≥0.5%/year)	0	0	0	Net Reclassification	0.135
		**Overall Reclassification (95% CI)**		**0.187 (0.043 – 0.331)**	
		**Continuous Reclassification (95% CI)**		**0.778 (0.568 – 0.987)**	

Supplemental Table 11: Net-reclassification analysis for cardiac death or myocardial infarction. The predicted risk from clinical data (rows) is compared to the risk from a model combining clinical and imaging data (columns). The clinical model included age, sex, and medical history. The combined model included clinical data as well as deep learning-derived imaging variables (coronary artery calcium, cardiac volumes, shape index, eccentricity index, and left ventricular mass index). Red shows patients reclassified to higher risk by one (light) or two (dark) categories, with blue showing patients recategorized to lower risk by one (light) or two (dark) categories. CI – confidence interval.

Supplemental Table 12

	EISNER<br>N=1240<br>30 (2.4%) CV death or MI	Low dose CT<br>N=913<br>32 (3.5%) death or MI
AUC		
Clinical	0.731 (0.658 – 0.802)	0.707 (0.613 – 0.801)
DL - Imaging	0.793 (0.727 – 0.858)	0.739 (0.642 – 0.835)
Combined model	0.838 (0.777 – 0.898)*	0.791 (0.709 – 0.872)*
Net Reclassification Index		
Categorical	0.148 (-0.082 to 0.378)	0.263 (0.067 – 0.460)
p-value	0.208	0.009
Continuous	0.233 (-0.127 to 0.592)	0.551 (0.210 – 0.892)
p-value	0.204	0.002

Supplemental Table 12: Risk stratification for cardiac events in young (age <60) non-smokers. In the Early Identification of Subclinical Atherosclerosis by Noninvasive Imaging Research (EISNER) we evaluated cardiac (CV) death or myocardial infarction (MI). In the low dose computed tomography (CT) population we evaluated death or MI. * - p<0.01 compared to clinical model using DeLong's test, AUC – area under the receiver operating characteristic curve, DL – deep learning.

Supplemental Table 13

	No events (N=3142)	Death or myocardial infarction (n=177)	p-value
Age	65 (56, 73)	68 (60, 77)	<0.001
Male	1746 (55.6%)	108 (61.0%)	0.160
Past Medical History			
Hypertension	1953 (62.2%)	107 (60.5%)	0.651
Diabetes	1009 (32.1%)	82 (46.3%)	<0.001
Dyslipidemia	1512 (48.1%)	87 (49.2%)	0.792
Family history of CAD	481 (15.3%)	12 (6.8%)	0.002
Smoking	506 (16.1%)	39 (22.0%)	0.038
CAC Category			<0.001
CAC 0	1293 (41.2%)	42 (23.7%)	
CAC 1-100	518 (16.5%)	37 (20.9%)	
CAC 101-400	460 (14.6%)	28 (15.8%)	
CAC>400	871 (27.7%)	70 (39.5%)	
LV volume (mL)	118.4 (97.0, 143.3)	129.4 (107.8, 160.9)	<0.001
LA volume (mL)	76.4 (62.4, 93.5)	85.0 (67.8, 105.4)	<0.001
RV volume (mL)	152.9 (124.4, 186.2)	156.7 (131.9, 189.9)	0.120
RA volume (mL)	78.3 (63.4, 98.2)	82.4 (63.2, 111.4)	0.161
LV mass (g)	138.3 (112.6, 168.4)	148.0 (123.3, 185.2)	<0.001
Shape Index	0.75 (0.70, 0.80)	0.76 (0.71, 0.81)	0.290
Eccentricity Index	0.75 (0.72, 0.79)	0.75 (0.70, 0.78)	0.070

Supplemental Table 13. Population characteristics for the cohort of patients undergoing low-dose chest CT for attenuation correction. Groups were compared using $\chi 2$ tests for categorical variables and Mann-Whitney U test for continuous variables. CAC – coronary artery calcification, CAD – coronary artery disease, LA – left atrium, LV – left ventricle, RA – right atrium, RV – right ventricle.

Supplemental Table 14

	Unadjusted HR (95% CI)	p-value	Adjusted HR (95% CI)	p-value
CAC 0	Reference		Reference	
CAC 1 – 100	2.03 (1.31 – 3.16)	0.002	1.68 (1.07 - 2.63)	0.023
CAC 101 – 400	1.91 (1.18 – 3.08)	0.008	1.44 (0.88 - 2.34)	0.148
CAC > 400	2.40 (1.64 – 3.52)	<0.001	1.56 (1.03 - 2.34)	0.034
Left ventricular volume (per 10 mL)	1.07 (1.04 – 1.11)	<0.001	--	
Right ventricular volume (per 10 mL)	1.03 (1.00 – 1.06)	0.062	0.96 (0.92 – 1.00)	0.041
Left atrial volume (per 10 mL)	1.10 (1.05 – 1.15)	<0.001	--	
Right atrial volume (per 10 mL)	1.04 (1.00 – 1.08)	0.076	--	
LV myocardial mass (per 10 g/m^2)	1.21 (1.14 – 1.30)	<0.001	1.29 (1.17 - 1.42)	<0.001
Shape index (per 0.1)	1.11 (0.90 – 1.36)	0.328	--	
Eccentricity index (per 0.1)	0.77 (0.69 – 1.01)	0.060	--	
Age (per 10 years)	1.31 (1.15 – 1.49)	<0.001	1.22 (1.06 - 1.41)	0.005
Male	1.23 (0.91 – 1.67)	0.172	--	
Hypertension	1.04 (0.77 – 1.40)	0.815	--	
Diabetes	1.66 (1.24 – 2.23)	0.001	1.51 (1.12 - 2.03)	0.007
Dyslipidemia	1.12 (0.83 – 1.50)	0.460	--	
Smoking	1.57 (1.10 – 2.24)	0.013	1.52 (1.06 - 2.18)	0.021
Family history	0.56 (0.31 – 1.00)	0.051	--	

Supplemental Table 14: Associations with cardiovascular death or myocardial infarction in patients undergoing low-dose chest CT for attenuation correction. Given the limited number of events, a parsimonious multivariable model was developed using backward stepwise elimination, until only variables with significant associations remained. Associations evaluated using Cox proportional hazards model, to account for multiple comparisons, p<0.001 is considered statistically significant. CAC – coronary artery calcium, CI – confidence interval, HR – hazard ratio.

Supplemental Table 15

Without Events	Low (<1%/year)	Intermediate (1-2%/year)	High (≥2%/year)		
Low (<1%/year)	595	100	13	Decreased Risk	0.260
Intermediate (1-2%/year)	515	832	256	Increased Risk	0.117
High (≥2%/year)	24	279	528	Net Reclassification	0.143
**With Events**	**Low (<1%/year)**	**Intermediate (1-2%/year)**	**High (≥2%/year)**		
Low (<1%/year)	12	5	3	Decreased Risk	0.144
Intermediate (1-2%/year)	9	41	33	Increased Risk	0.228
High (≥2%/year)	0	17	60	Net Reclassification	0.083
		**Overall Reclassification (95% CI)**		**0.211 (0.120 – 0.301)**	
		**Continuous Reclassification (95% CI)**		**0.334 (0.184 – 0.485)**	

Supplemental Table 15: Net-reclassification analysis for death or myocardial infarction. The predicted risk from clinical data (rows) are compared to the risk from a model combining clinical and imaging data (columns). The clinical model included age, sex, and medical history. The combined model included clinical data as well as deep learning derived imaging variables (coronary artery calcium, cardiac volumes, shape index, eccentricity index, and left ventricular mass index). Red shows patients reclassified to higher risk by one (light) or two (dark) categories, with blue showing patients recategorized to lower risk by one (light) or two (dark) categories. CI – confidence interval.

Supplemental Table 16

LV Volume	Normal baseline	Abnormal baseline
Normal follow-up	21997	59
Abnormal follow-up	52	78
RV Volume	Normal baseline	Abnormal baseline
Normal follow-up	21947	64
Abnormal follow-up	79	96
LA Volume	Normal baseline	Abnormal baseline
Normal follow-up	21845	66
Abnormal follow-up	113	162
RA Volume	Normal baseline	Abnormal baseline
Normal follow-up	22040	28
Abnormal follow-up	58	60
LV mass index	Normal baseline	Abnormal baseline
Normal follow-up	17792	599
Abnormal follow-up	1616	2285

Supplemental Table 16: Comparison of patient classification at baseline and follow-up imaging. LA – left atrium, LV – left ventricle, RA – right atrium, RV – right ventricle.
