# Supplementary material for: Predicting mortality from AI cardiac volumes mass and coronary calcium on chest computed tomography
Source: Nat Commun. 2024 Mar 29;15:2747. doi: 10.1038/s41467-024-46977-3 (PMC10980695; doi:10.1038/s41467-024-46977-3)
Supplement: Supplementary file 3 — Reporting Summary [file 41467_2024_46977_MOESM3_ESM.pdf]

nature portfolio

Corresponding author(s): Piotr Slomka

Last updated by author(s): 03/08/2024

Reporting Summary

Nature Portfolio wishes to improve the reproducibility of the work that we publish. This form provides structure for consistency and transparency in reporting. For further information on Nature Portfolio policies, see our Editorial Policies and the Editorial Policy Checklist.

Statistics

For all statistical analyses, confirm that the following items are present in the figure legend, table legend, main text, or Methods section.

n/a	Confirmed	
☐	☒	The exact sample size (*n*) for each experimental group/condition, given as a discrete number and unit of measurement
☐	☒	A statement on whether measurements were taken from distinct samples or whether the same sample was measured repeatedly
☐	☒	The statistical test(s) used AND whether they are one- or two-sided<br>*Only common tests should be described solely by name; describe more complex techniques in the Methods section.*
☐	☒	A description of all covariates tested
☐	☒	A description of any assumptions or corrections, such as tests of normality and adjustment for multiple comparisons
☐	☒	A full description of the statistical parameters including central tendency (e.g. means) or other basic estimates (e.g. regression coefficient) AND variation (e.g. standard deviation) or associated estimates of uncertainty (e.g. confidence intervals)
☐	☒	For null hypothesis testing, the test statistic (e.g. *F*, *t*, *r*) with confidence intervals, effect sizes, degrees of freedom and *P* value noted<br>*Give P values as exact values whenever suitable.*
☒	☐	For Bayesian analysis, information on the choice of priors and Markov chain Monte Carlo settings
☒	☐	For hierarchical and complex designs, identification of the appropriate level for tests and full reporting of outcomes
☐	☒	Estimates of effect sizes (e.g. Cohen's *d*, Pearson's *r*), indicating how they were calculated

Our web collection on statistics for biologists contains articles on many of the points above.

Software and code

Policy information about availability of computer code

Data collection	No specific software was used for clinical data collection, which was retrieved from three separate cohorts as outlined in detail in the manuscript.
Data analysis	All analyses were performed using Stata/IC version 13.1 (StataCorp, College Station, Texas, USA) and R (version 4.1.2) including the "DAGitty" package.

For manuscripts utilizing custom algorithms or software that are central to the research but not yet described in published literature, software must be made available to editors and reviewers. We strongly encourage code deposition in a community repository (e.g. GitHub). See the Nature Portfolio guidelines for submitting code & software for further information.

Data

Policy information about availability of data

All manuscripts must include a data availability statement. This statement should provide the following information, where applicable:

- Accession codes, unique identifiers, or web links for publicly available datasets
- A description of any restrictions on data availability
- For clinical datasets or third party data, please ensure that the statement adheres to our policy

All derived data supporting the findings of this study are available within the paper, in the supplementary information file, and in the source data file. Original data from the NLST can be requested through the National Cancer Institute. Restricted access for the deidentified EISNER, and low-dose CT populations can be obtained

via requests to the corresponding author Dr. Piotr Slomka. Requests should include the name and contact details of the person requesting the data, which data and clinical variables are requested and the purpose of requesting the data. Requests will be subject to consideration by the steering committees of the cohorts and the investigational review board of Cedars-Sinai Medical Center and investigational review boards from other centers if applicable. Time frame for a response will be within 3 months. Data requests under agreement will be considered for the purpose of reproducing the data and subject to appropriate confidentiality obligations and restrictions. Source data are provided with this paper.

Research involving human participants, their data, or biological material

Policy information about studies with human participants or human data. See also policy information about sex, gender (identity/presentation), and sexual orientation and race, ethnicity and racism.

Reporting on sex and gender	We utilized sex-specific normal limits and incorporated sex as a biologic variable throughout the analysis.
Reporting on race, ethnicity, or other socially relevant groupings	Neither race nor ethnicity were included in our analysis. The majority of patients in the NLST trial identify as white (91% in our cohort) and we do not have information regarding either race or ethnicity in the remaining two populations. We have included the following limitation:<br>We did not incorporate race or ethnicity into our analyses. The majority of patients in the NLST trial were white (91% in our cohort); future studies should evaluate methods to incorporate more diverse populations.
Population characteristics	We included a total of 24354 patients with median age 61 (IQR 57 – 65), of whom and 14441 (59.3%) were males. Additional details for the populations have been outlined in Table 1, Supplemental Table 9, and Supplemental Table 11.
Recruitment	The study used de-identified image sets and did not collect new data, therefore the research is considered non-human subject research. We included subjects from 3 separate external testing cohorts including patients from the NLST (NCT00047385), a multicenter randomized controlled trial of patients randomized to low-dose chest CT for lung cancer screening, asymptomatic patients from the EISNER trial (NCT00927693) who underwent CAC scanning, and patients from two centers who underwent myocardial perfusion imaging with low-dose, ungated, chest CT for attenuation correction. The NLST trial included current or former heavy smokers between the ages of 55 and 74. Patients were randomly assigned to non-contrast, non-ECG-gated chest CT imaging between 2002 and 2007. We included 24805 subjects from this external cohort (previously unseen by the DL models) with available baseline CT imaging and follow-up for mortality. Of those, we excluded cases where image files were corrupt (n=133, 0.5%), the scan length was less than 12cm or did not include the heart (n=292, 1.2%), and cases where segmentation failed (n=26, 0.1%), leaving 24354 subjects. In cases where segmentation failed, neither the CAC nor the cardiac volume model was able to process the scan. The baseline CT was used to assess associations with outcomes. We also compared estimates from baseline CT scans with estimates from CT scans performed at 1 year in 22292 patients as a measure of stability. Lastly, we compared cardiac volumes and left ventricular mass in a cohort of 80 patients from a clinical trial. These patients underwent low-dose, ungated CT and contrast-enhanced, ECG-gated, cardiac CT angiography on the same day, during a single imaging session, minimizing potential differences between scans (NCT02110303).
Ethics oversight	The study protocol complied with the Declaration of Helsinki and was approved by the institutional review boards at participating institutions. For the EISNER (NCT00927693) trial, the original study was reviewed and approved by the institutional review board at Cedars-Sinai (IRB#3351). For the DIAMOND (NCT02110303) trial, the original study was reviewed and approved by the Scottish Research Ethics Committee (REC reference: 14/SS/0089). For the NLST trial (NCT00047385) trial, the original study was reviewed and approved by the 33 participating institutions. The remaining cohorts were collected through the REFINE-SPECT registry which was reviewed and approved by the institutional review board at Cedars-Sinai Medical Center (IRB# 19604). The study used de-identified image sets and did not collect new data, therefore the research is considered non-human subject research.

Note that full information on the approval of the study protocol must also be provided in the manuscript.

Field-specific reporting

Please select the one below that is the best fit for your research. If you are not sure, read the appropriate sections before making your selection.

☒ Life sciences ☐ Behavioural & social sciences ☐ Ecological, evolutionary & environmental sciences

For a reference copy of the document with all sections, see nature.com/documents/nr-reporting-summary-flat.pdf

Life sciences study design

All studies must disclose on these points even when the disclosure is negative.

Sample size	We included all possible patients from the NLST, EISNER, and DIAMOND trials. We included all available patients with CT information from the REFINE study at the time of the analysis.
Data exclusions	For the NLST trial, we included 24805 subjects from this external cohort (previously unseen by the DL models) with available baseline CT imaging and follow-up for mortality. Of those, we excluded cases where image files were corrupt (n=133, 0.5%), the scan length was less than 12cm or did not include the heart (n=292, 1.2%), and cases where segmentation failed (n=26, 0.1%). Those patients were excluded because the variables of interest (CAC, LV mass, cardiac volumes) could not be evaluated. All scans from the EISNER and DIAMOND trials were included.
Replication	We included two external populations for validation. We identified similar associations with outcomes in these populations.

Randomization	This is not applicable. We were evaluating the prognostic importance of cardiac chamber measurements and coronary artery calcium which are biologic measurements and cannot be randomized.
Blinding	All image analysis was performed blinded to clinical outcomes or patient characteristics. We utilized previously collected images, which were obtained prior to clinical events occurring.

Reporting for specific materials, systems and methods

We require information from authors about some types of materials, experimental systems and methods used in many studies. Here, indicate whether each material, system or method listed is relevant to your study. If you are not sure if a list item applies to your research, read the appropriate section before selecting a response.

Materials & experimental systems

n/a	Involved in the study
☒	☐ Antibodies
☒	☐ Eukaryotic cell lines
☒	☐ Palaeontology and archaeology
☒	☐ Animals and other organisms
☐	☒ Clinical data
☒	☐ Dual use research of concern
☒	☐ Plants

Methods

n/a	Involved in the study
☒	☐ ChIP-seq
☒	☐ Flow cytometry
☒	☐ MRI-based neuroimaging

Clinical data

Policy information about clinical studies

All manuscripts should comply with the ICMJE guidelines for publication of clinical research and a completed CONSORT checklist must be included with all submissions.

Clinical trial registration	NCT00047385; NCT00927693; and NCT02110303
Study protocol	Full trial protocols have been previously published. The relevant publications are:<br>National Lung Screening Trial. The National Lung Screening Trial: overview and study design. Radiology 258, 243-253 (2011).<br>Rozanski, A., et al. Impact of coronary artery calcium scanning on coronary risk factors and downstream testing the EISNER prospective randomized trial. J Am Coll Cardiol 57, 1622-1632 (2011).<br>Joshi NV et al. 18F-fluoride positron emission tomography for identification of ruptured and high-risk coronary atherosclerotic plaques: a prospective clinical trial.Lancet. 2014; 383:705–713. doi: 10.1016/S0140-6736(13)61754-7
Data collection	We included subjects from 3 separate external testing cohorts including patients from the NLST (NCT00047385), a multicenter randomized controlled trial of patients randomized to low-dose chest CT for lung cancer screening34, asymptomatic patients from the EISNER trial (NCT00927693) who underwent CAC scanning, and patients from two centers who underwent myocardial perfusion imaging with low-dose, ungated, chest CT for attenuation correction. The NLST trial included current or former heavy smokers between the ages of 55 and 74. Patients were randomly assigned to non-contrast, non-ECG-gated chest CT imaging between 2002 and 2007. We included 24805 subjects from this external cohort (previously unseen by the DL models) with available baseline CT imaging and follow-up for mortality. The EISNER trial randomized patients to CAC scanning or risk factor modification without scanning. CAC scans were collected at Cedars-Sinai Medical Center between May 2001 and May 2005, followed for death or non-fatal myocardial infarction using electronic medical records. For the DIAMOND trial, patients were recruited from the Royal Infirmary of Edinburgh underwent non-contrast CT and contrast-enhanced gated CT during a single imaging session between February 2012 and January 2013.
Outcomes	In the NLST trial population, the primary outcome was cardiovascular mortality and all-cause mortality was a secondary outcome. Patients had follow-up for all-cause mortality and information from death certificates regarding underlying cause. Cardiovascular mortality was determined for the ICD-10 codes using established definitions for cardiovascular mortality 36, and validated ICD-10 codes. For the EISNER population, medical history was determined at baseline and patients were followed prospectively for occurrence of cardiovascular death or myocardial infarction. For the third external population, demographics and medical history were determined at the time of CT scanning and incidence of all-cause mortality of myocardial infarction was determined from administrative databases. For the DIAMOND trial, we evaluated agreement between cardiac chamber measurements from ungated, non-contrast CT with contrast-enhanced, gated CT.

Plants

Seed stocks	n/a
Novel plant genotypes	n/a
Authentication	n/a
